# Higher dietary acid load is associated with hyperuricemia in Chinese adults: a case-control study

**DOI:** 10.1186/s12902-022-01192-3

**Published:** 2022-11-18

**Authors:** Si-si Shao, Chong-ze Lin, Yong-fu Zhu, Chun Chen, Qian-jia Wu, Ruo-ru Chen

**Affiliations:** 1 Department of nephropathy rheumatology and immunology, Wenzhou hospital of traditional Chinese medicine affiliated to Zhejiang Chinese medicine university, Wenzhou City, China; 2 Department of nephropathy rheumatology and immunology, Wenzhou hospital of traditional Chinese medicine affiliated to Zhejiang Chinese medicine university, No. 9, Liuhongqiao Road, Lucheng District, Wenzhou City, 325000 China

**Keywords:** Dietary acid load, Hyperuricemia, Chinese, Case-control

## Abstract

**Background:**

This study aims to explore the association between dietary acid load and hyperuricemia in Chinese adults.

**Methods:**

A case-control study was conducted. Adult participants with hyperuricemia were recruited as the cases and those without hyperuricemia were as the controls. Food consumption was evaluated by food frequency questionnaire (FFQ). Dietary acid load was assessed by potential renal acid load (PRAL) and net endogenous acid production (NEAP). Dietary acid load was divided into four levels: the first quartile (Q1), the second quartile (Q2), the third quartile (Q3) and the fourth quartile (Q4). Logistic regression model was applied for exploring the association between dietary acid load (PRAL and NEAP) and hyperuricemia. Odds ratio (OR) and its correspondence confidence interval (CI) were computed.

**Results:**

A total of 290 participants were eligible in this study, in which there were 143 individuals in case group and 147 in control group. A higher level of PRAL was found to be associated with odds of hyperuricemia. ORs of hyperuricemia for Q2, Q3 and Q4 of PRAL were 2.74 (95%CI: 1.94 ~ 3.88, *p*-value: 0.004), 2.90 (95%CI: 2.05 ~ 4.10, *p*-value: 0.002) and 3.14 (95%CI: 2.22 ~ 4.45, *p*-value: 0.001), respectively. There was a positive association between elevated NEAP and hyperuricemia. OR of hyperuricemia for Q2 was not material significance (OR:1.54, 95%CI: 0.93 ~ 2.53, *p*-value: 0.210), however, ORs of hyperuricemia for Q3 (OR: 2.40, 95%CI: 1.70 ~ 3.38, *p*-value: 0.011) and Q4 (OR: 3.27, 95%CI: 2.31 ~ 4.62, *p*-value: 0.001) were statistically significant.

**Conclusion:**

Higher level of dietary acid load was found to be associated with hyperuricemia in Chinese adults, indicative of advocation of a well-balanced diet in this population.

## Introduction

Hyperuricemia is characterized by abnormally high serum uric acid, and it may lead to deposition of sodium urate crystal, especially in arthrosis, if without any treatments [1]. The long-term accumulation of urate crystal might cause harm to the joint structure and bring huge aching feeling to the patients, which affect their life quality [2, 3]. Previously studies also reported a higher risk of chronic diseases in patients with hyperuricemia, such as cardiovascular disease, diabetes, kidney disease etc. [4–6] In addition, increasing evidence showed that a higher prevalence of hyperuricemia was associated with a higher all-cause mortality [7–11]. The impacts of hyperuricemia on general population in some regions have been underestimated and ignored [12].

Prevalence of hyperuricemia varies in different ethnic groups, countries and territories, and it shows a positive correlation between economic growth and the prevalence of hyperuricemia [12]. A prevalence rate of 21.4% in the American adults was reported according to the Health and Nutrition Examination Survey in a period between 2007 and 2008 [13]. When it comes to China, 13.3% of the total population were found to have abnormally high serum uric acid from 2000 to 2014 [14]. More recently, two Chinese nationally representative cross-sectional surveys demonstrated a higher rate in men (24.4%) than women (3.6%) in 2018 ~ 2019 [15], which indicates that men are more susceptible to hyperuricemia than women.

A variety of factors contribute to the long-run increase of hyperuricemia in Chinese adults, and diet pattern modification in the last decades might be the most important one [16, 17]. The consumption of animal source food has been increased sharply in last a few years, especially livestock and poultry meat, animal fishery products etc., which contain rich purine [16, 17]. As uric acid is the final product of purine metabolism, an increased risk of hyperuricemia in people with higher animal source food consumption was documented [18]. However, some studies suggest that a higher consumption of fruit and vegetable was associated with a lower risk of hyperuricemia [19, 20]. In order to comprehensively assess effect of consuming different food group, dietary acid load was brought up [21]. Acid precursors and base precursors from various kinds of consuming food are evaluated [21]. Among the compute methods of dietary acid load, potential renal acid load (PRAL) and net endogenous acid production (NEAP) were widely applied [21]. Chronic acid-base imbalance, especially high dietary acid load, has been found to be associated with several kinds of diseases, such as hypertension, diabetes, cancer etc. [21–25], while its impact on hyperuricemia is not fully understood. This study based on case-control design aims to explore the association between dietary acid load and hyperuricemia in Chinese adults.

## Methods

### Study design and participants

This study was based on case-control design. Participants were recruited from Wenzhou hospital of traditional Chinese medicine affiliated to Zhejiang Chinese medicine university in a period from January 2017 to December 2021. Participants diagnosed with hyperuricemia were included in case group and should also meet the following criteria: (1) with an age > 18 years; (2) with basic communication capability and memory; (3) without cancer, neuropsychological diseases, diabetes and gastrointestinal diseases, as these diseases have large impact on food pattern; (4) the food pattern was not largely changed in last 12 months. Almost the same number of participants without hyperuricemia were included in the control group. Male proportion and age distribution in the control group were similar to the case group, while other eligible criteria for the control group were also in accord with the case group. This study was performed in line with the principles of the Declaration of Helsinki, and approved by the Ethical Committee of Wenzhou hospital of traditional Chinese medicine affiliated to Zhejiang Chinese medicine university. Informed consent was obtained from all individual participants included in the study.

Anthropological data and health-related information were derived from electronic patient record. Anthropological data included age, gender, weight (kg), height (cm) and body mass index (BMI). Health-related information included diastolic blood pressure (mmHg), systolic blood pressure (mmHg), heart rate (beats/minutes), serum uric acid (μmol/L), smoking, drinking and chronic diseases. Smoking statues were divided into current smoking, ever smoking and never smoking. Ever smoking was defined as those who quit smoking for more than 12 months. Alcohol drinking statues were classified as current drinking, ever drinking and never drinking. Ever drinking was defined as those who quit drinking for more than 12 months. The list of chronic diseases was as follows: hypertension, coronary heart disease, osteoporosis, chronic obstructive pulmonary disease， chronic kidney disease and hyperlipidemia. These chronic diseases required a clear clinical diagnosis, and self-reporting was not considered in this study as the lack of uniform standards of diseases assessment from different participants.

### Definition of hyperuricemia

We used a Chinese general criterion to diagnose hyperuricemia in this study. Hyperuricemia for men was defined as fasting serum uric acid > 420 μmol/L in two separate days, and for women was > 360 μmol/L. [26] In addition, individuals diagnosed as gout or underwent hyperuricemia treatment were also classified as hyperuricemia.

### Food consumption survey by food frequency questionnaire

A validated food frequency questionnaire (FFQ) was applied for food consumption survey. Briefly, FFQ in this study was adapted from China Health and Nutrition Survey which is an ongoing open cohort and designed to examine the effects of the health, nutrition status and other factors on the study population [27]. Given the differences in local diet pattern, 12 representative food groups in Zhejiang province were set in the FFQ, inclusive of grains, potato, vegetable, fruit, soy, fish, red meat, poultry, dairy, egg, beverages and edible oil. A small-scale pre-survey was also conducted to verify FFQ validity. Food consumption survey was performed though face-to-face interview by well-trained investigators. The participants were asked to answer diet-related information in a period of last 12 months. Eating amount (g/time or ml/time) and eating frequency (times/day, times/week, times/month or times/year) were recorded. Daily consumption of the 12 food groups were calculated, respectively.

### Food consumption and dietary acid load

In order to compute daily nutrient intake, the related data from the Chinese food composition table (2012 version) were used. Energy intake and three macro-nutrients including carbohydrate, fat and protein were calculated. In order to evaluate dietary acid load (PRAL and NEAP), additional nutrients including sodium, magnesium, phosphorus, magnesium and calcium were also computed. According to previously studies [21], PRAL and NEAP were calculated by the following formulas, respectively:

1. PRAL (mEq/d) =0.4888 × protein intake (g/d) + 0.0366 × phosphorus (mg/d) - 0.0205 × potassium (mg/d) - 0.0125 × calcium (mg/d) - 0.0263 × magnesium (mg/d);
2. NEAP (mEq/d) = (54.5 × protein intake (g/d) ÷ potassium intake (mEq/d))-10.2

### Statistical analysis

Variables on discrete distribution were as number and proportion, while if variables on continuous distribution were in accord with normal distribution than they were exhibited as mean and standard deviation (SD), otherwise were exhibited as median and range interquartile (IQR). Variable difference was evaluated by χ^2^ test, student’s test or Wilcoxon rank sum test. Logistic regression model was applied to assess the association between dietary acid load (PRAL and NEAP) and the odds of hyperuricemia. Dietary acid load was divided into four level: the first quartile (Q1), the second quartile (Q2), the third quartile (Q3), and the fourth quartile (Q4), in which each group has an approximately same number of participants. Q1 was considered as a reference group and odd ratios (ORs) and the correspondent confidence intervals (CIs) of Q2, Q3 and Q4 were computed. Additionally, possible risk factors, such as age and sex, as well as multiply variables (age, sex, BMI, diastolic blood pressure, systolic blood pressure, smoking, drinking and chronic disease) were put in the Logistic regression model to obtain adjusted effects. Statistical analysis was conducted in R language (version 3.5.1, R Core Development Team). Forest plots were produced based on R language software package named FORESTPLOT. In this study, a *p*-value < 0.05 was perceived as statistical significance.

## Results

### Characteristic of participants

**Fig. 1:**
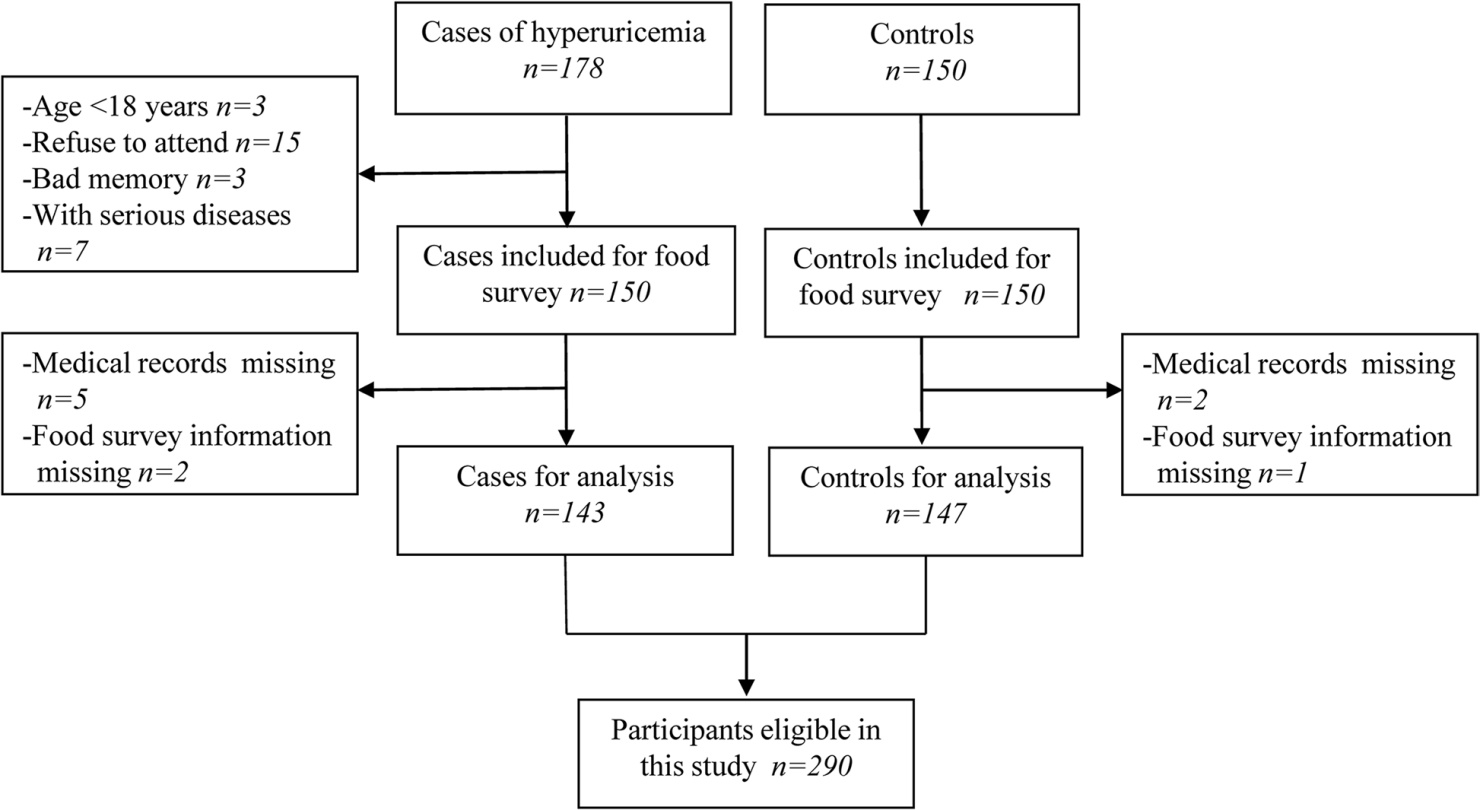
Flow chart of participants selection

A total of 290 participants were eligible in this study, in which there were 143 cases (diagnosed with hyperuricemia) and 147 controls (without hyperuricemia). Participant selection was exhibited in flowing chart (Fig. 1). In the case group, male participant was accounting for 53.85 and 55.78% in control group. An average age in the case group was 43.29 ± 12.93 years and 42.91 ± 10.44 years in the control group. Except for serum uric acid, other baseline variables including age, male proportion, weight, height, BMI, diastolic blood pressure, systolic blood pressure, heart rate, smoking, drinking and chronic disease, were lack of material significance between participants in case group and control group (*p*-value < 0.05). Characteristic of participants was summarized in Table 1.

**Table 1:** Baseline information of participants in case group and control group

Variates	Case group (*n* = 143)		Control group (*n* = 147)		*p*-value
	Mean	SD	Mean	SD	
Age (year)	43.29	12.93	42.91	10.44	0.782
Male (N, %)	77	53.85	82	55.78	0.741
Weight (kg)	66.06	11.97	64.06	11.70	0.151
Height (cm)	162.40	8.65	163.11	8.14	0.472
BMI	25.01	3.81	24.25	3.44	0.076
Diastolic blood pressure (mmHg)	122.60	16.07	119.19	16.13	0.072
Systolic blood pressure (mmHg)	75.98	11.04	74.55	11.30	0.276
Heart rate (beats/minutes)	76.79	11.07	77.15	11.20	0.782
Serum uric acid (μmol/L)	467.78	84.21	328.24	58.13	< 0.001
Smoking (N, %)					0.196
Current	41	28.67	29	19.73	
Ever	8	5.59	8	5.44	
Never	94	65.73	110	74.83	
Drinking (N, %)					0.834
Current	42	29.37	43	29.25	
Ever	18	12.59	22	14.97	
Never	83	58.04	82	55.78	
Chronic diseases (N, %)					0.081
No	93	65.03	101	68.71	
1 ~ 2	48	33.57	38	25.85	
3~	2	1.40	8	5.44	

### Food consumption, nutrients intake and dietary acid load

As respect to food groups, vegetable consumption and fruit consumption in the case group were material lower than in the control group (*p*-value < 0.05). Fish consumption in case group were material higher than in control group (*p*-value < 0.05). In terms of other food groups including grains, potato, soy, red meat, poultry, dairy, egg, beverages and edible oil, there were lack of significant differences between participants in case group and control group, shown in Table 2.

**Table 2:** Comparison of food consumption, nutrients intake and dietary acid load between case group and control group

Variates	Case group (*n* = 143)		Control group (*n* = 147)		*p*-value
	Median	IQR	Median	IQR	
Food groups (g/d)					
Grains	150.00	100.00 ~ 205.00	150.00	100.00 ~ 200	0.290
Potato	5.00	0.19 ~ 19.33	6.67	1.04 ~ 17.67	0.345
Vegetable	240.00	160.00 ~ 400.00	300.00	200.00 ~ 500.00	0.028
Fruit	100.00	42.86 ~ 220.00	128.08	60.87 ~ 242.61	0.035
Soy	0.27	0 ~ 1.52	0.33	0 ~ 1.55	0.987
Fish	47.38	0 ~ 120.00	31.43	0 ~ 86.40	0.026
Red meat	100.00	50.00 ~ 160.00	92.00	28.57 ~ 146.43	0.181
Poultry	14.29	7.14 ~ 37.86	16.43	5.52 ~ 28.57	0.376
Dairy	5.00	0 ~ 14.00	6.08	0 ~ 17.00	0.231
Egg	21.43	12.14 ~ 50.00	23.57	14.29 ~ 50.00	0.585
Beverages	0.00	0 ~ 40.00	0.00	0 ~ 58.33	0.719
Edible oil	26.67	20.00 ~ 33.33	23.81	13.89 ~ 33.33	0.082
Nutrient intake					
Energy (kcal/d)	1469.53	1170.3 ~ 1798.79	1426.26	1132.24 ~ 1706.09	0.323
Carbohydrate (g/d)	154.74	119.88 ~ 215.82	158.56	119.05 ~ 208.11	0.725
Fat (g/d)	63.65	49.41 ~ 80.59	60.25	43.43 ~ 76.45	0.133
Protein (g/d)	61.56	46.31 ~ 86.53	57.43	39.36 ~ 84.89	0.159
Sodium (mg/d)	1466.25	1208.42 ~ 1995.68	1413.93	1127.72 ~ 1822.13	0.171
Magnesium (mg/d)	254.39	204.28 ~ 316.45	249.64	190.73 ~ 340.86	0.840
Phosphorus (mg/d)	925.05	769.26 ~ 1293.56	938.20	692.72 ~ 1292.44	0.464
Potassium (mg/d)	1732.09	1430.68 ~ 2282.74	1821.88	1297.71 ~ 2451.89	0.766
Calcium (mg/d)	431.90	294.87 ~ 633.28	416.40	286.79 ~ 615.97	0.765
Dietary acid load (mEq/d)					
PRAL	16.99	7.39 ~ 28.65	11.82	2.84 ~ 22.24	0.003
NEAP	67.30	51.15 ~ 79.26	56.69	43.32 ~ 71.67	< 0.001

Abbreviation: *IQR* Range interquartile, *PRAL* Potential renal acid load, *NEAP* Net endogenous acid production

As for nutrients intake including carbohydrate, fat, protein, sodium, magnesium, phosphorus, magnesium and calcium, there were lack of statistically significant difference between participants in case group and control group, shown in Table 2.

**Fig. 2:**
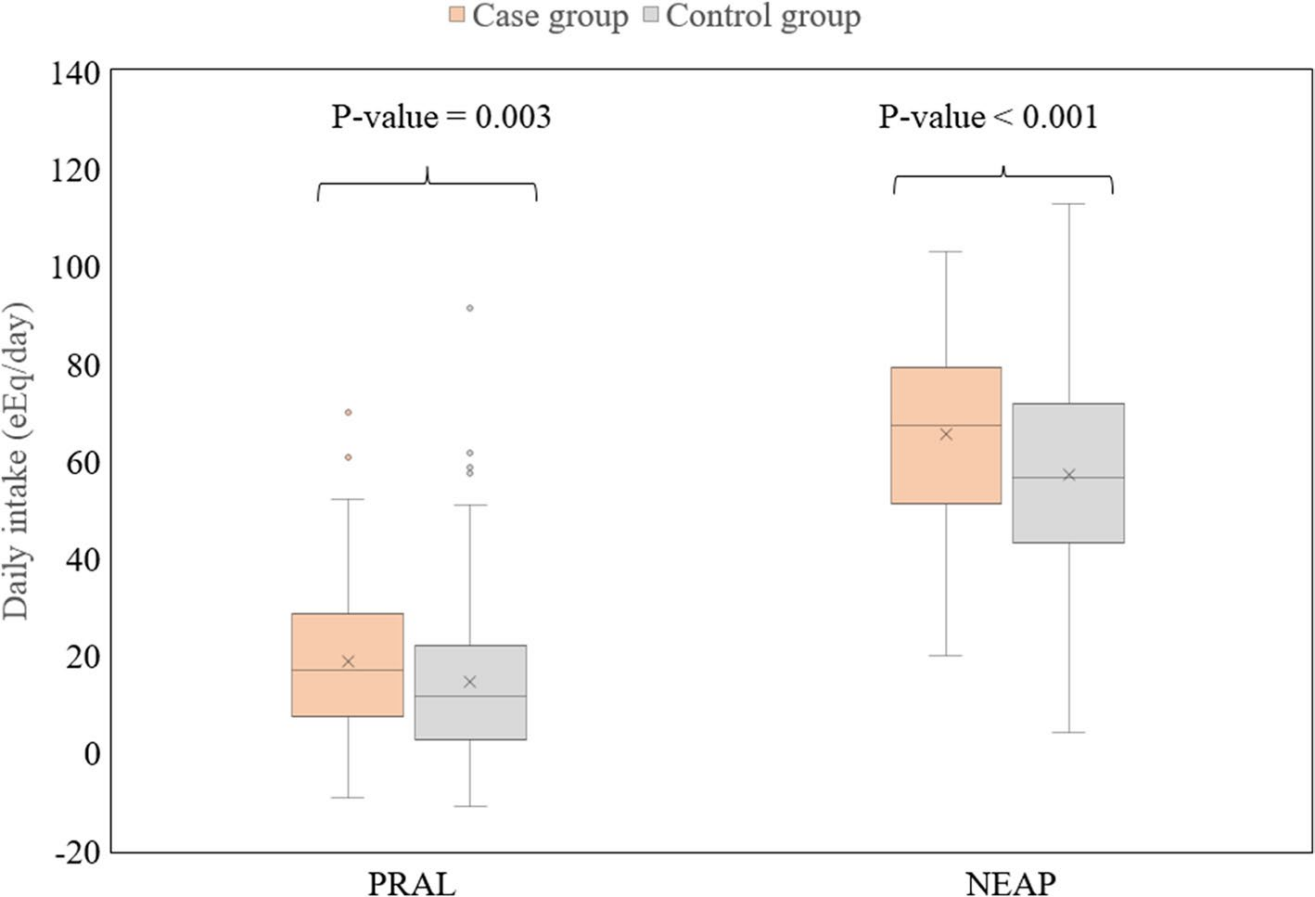
Comparison of PRAL and NEAP between case group and control group

PRAL in the case group was significantly higher than the control group. In addition, NEAP in the case group was also higher than in the control group, shown in Table 2 and Fig. 2.

### The association between PRAL and hyperuricemia

**Fig. 3:**
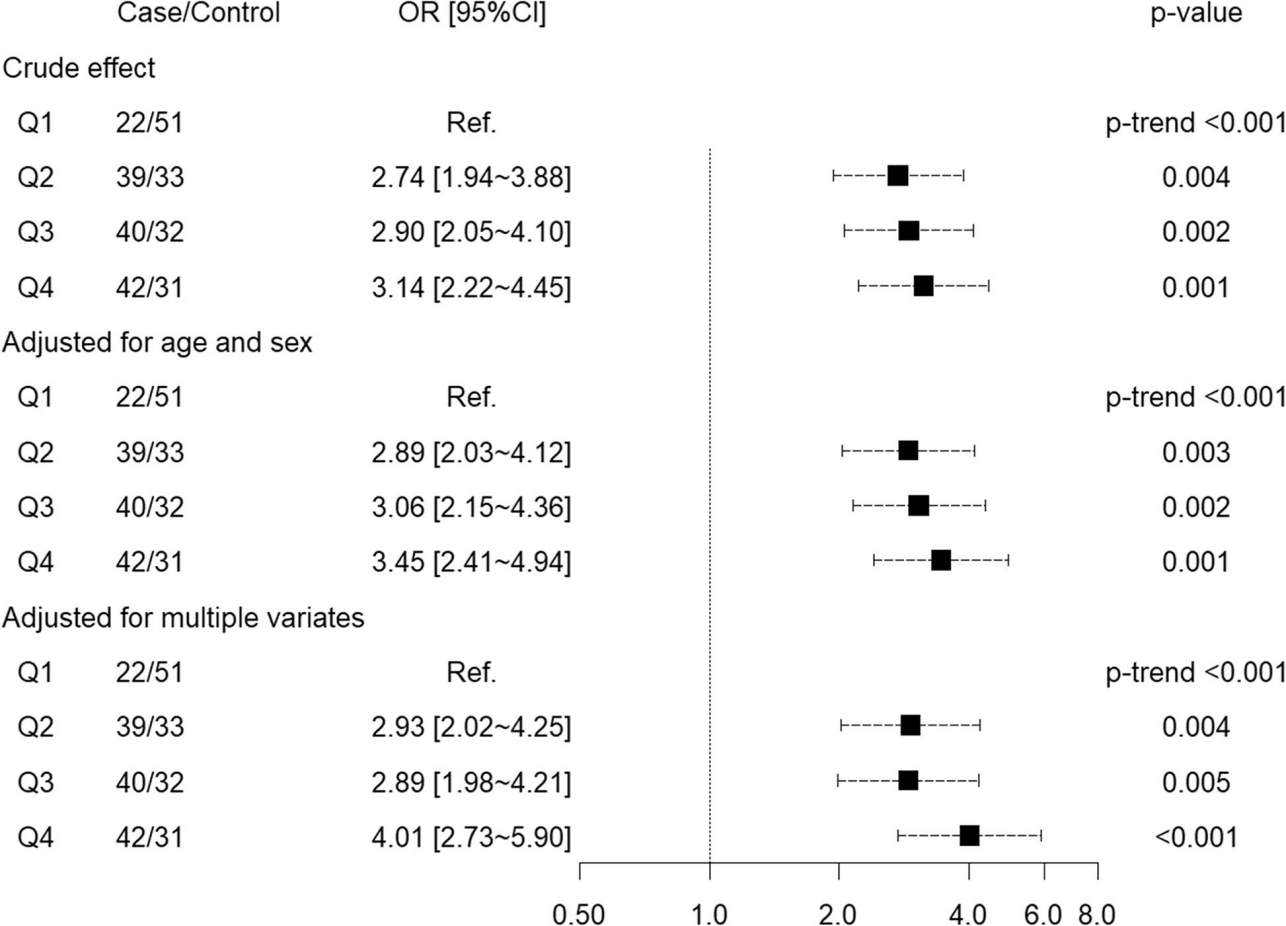
Forest plot about odd ratio of hyperuricemia in different groups according to PRAL

Higher PRAL was found to be associated with hyperuricemia. Crude ORs of hyperuricemia for Q2, Q3 and Q4 of PRAL were 2.74 (95%CI: 1.94 ~ 3.88, *p*-value: 0.004), 2.90 (95%CI: 2.05 ~ 4.10, *p*-value: 0.002) and 3.14 (95%CI: 2.22 ~ 4.45, *p*-value: 0.001), respectively and *p*-value for trend was < 0.001. The positively significant associations were remained after adjusting for age and sex, or for multiple variables, shown in Table 3 and Fig. 3.

**Table 3:** Odds ratio of hyperuricemia in different groups according to PRAL

Variates	Q1	Q2	Q3	Q4
PRAL (mEq/d)	−10.94~	3.76~	12.83~	24.80~
n	73	72	72	73
Case group	22	39	40	42
Control group	51	33	32	31
Crude effect				
OR	Ref.	2.74	2.90	3.14
95% CI	–	(1.94 ~ 3.88)	(2.05 ~ 4.10)	(2.22 ~ 4.45)
*p*-value	–	0.004	0.002	0.001
*p*-trend	< 0.001			
Adjusted for age and sex				
OR	Ref.	2.89	3.06	3.45
95% CI	–	(2.03 ~ 4.12)	(2.15 ~ 4.36)	(2.41 ~ 4.94)
*p*-value	–	0.003	0.002	0.001
*p* for trend	< 0.001			
Adjusted for multiple variates*				
OR	Ref.	2.93	2.89	4.01
95% CI	–	(2.02 ~ 4.25)	(1.98 ~ 4.21)	(2.73 ~ 5.90)
*p*-value	–	0.004	0.005	< 0.001
*p* for trend	< 0.001			

Abbreviation: *PRAL* Potential renal acid load, *OR* Odd ratio, *CI* Confidence interval, *Q1* the First quartile, *Q2* the Second quartile, *Q3* the Third quartile, *Q4* the Fourth quartile. *Multiple variates included age, sex, BMI, diastolic blood pressure, systolic blood pressure, smoking, drinking and chronic disease

### Association between NEAP and hyperuricemia

**Fig. 4:**
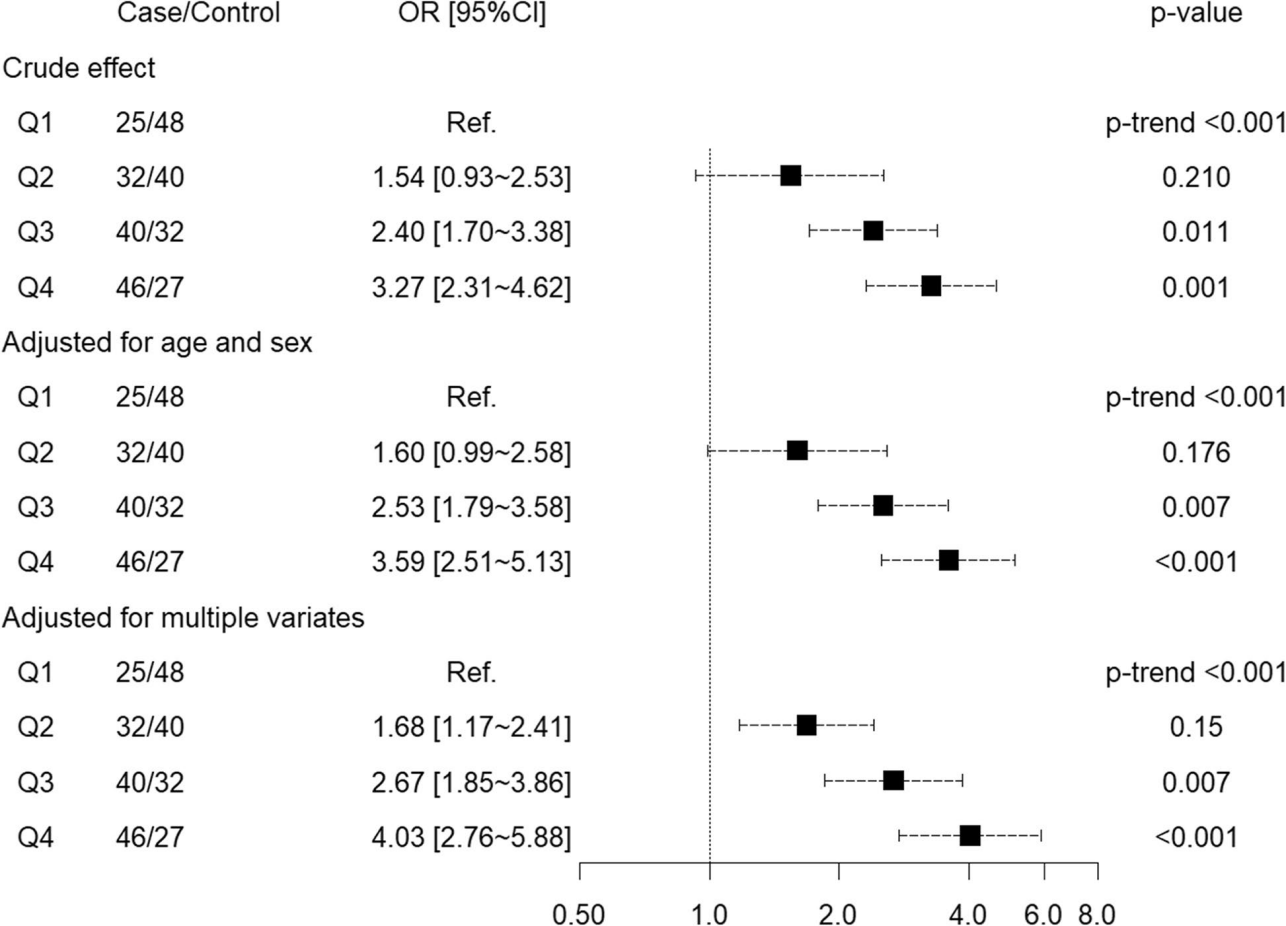
Forest plot about odd ratio of hyperuricemia in different groups according to NEAP

There was a positive association between elevated NEAP and hyperuricemia. Crude OR of hyperuricemia for Q2 was not material significance (OR:1.54, 95%CI: 0.93 ~ 2.53, *p*-value: 0.210), however, ORs of hyperuricemia for Q3 (OR: 2.40, 95%CI: 1.70 ~ 3.38, *p*-value: 0.011) and Q4 (OR: 3.27, 95%CI: 2.31 ~ 4.62, *p*-value: 0.001) were statistically significant. *P*-value for trend was < 0.001 indicative of a dose-response relationship between NEAP and hyperuricemia. After adjusting for age and sex, or for multiple variables, the positive associations did not fluctuate largely, shown in Table 4 and Fig. 4.

**Table 4:** Odds ratio of hyperuricemia in different groups according to NEAP

Variates	Q1	Q2	Q3	Q4
NEAP (mEq/d)	4.14~	44.76~	59.22~	76.01~
n	73	72	72	73
Case group	25	32	40	46
Control group	48	40	32	27
Crude effect				
OR	Ref.	1.54	2.40	3.27
95% CI	–	(0.93 ~ 2.53)	(1.7 ~ 3.38)	(2.31 ~ 4.62)
*p*-value	–	0.210	0.011	0.001
*p* for trend	< 0.001			
Adjusted for age and sex				
OR	Ref.	1.60	2.53	3.59
95% CI	–	(0.99 ~ 2.58)	(1.79 ~ 3.58)	(2.51 ~ 5.13)
*p*-value	–	0.176	0.007	< 0.001
*p* for trend	< 0.001			
Adjusted for multiple variates				
OR	Ref.	1.68	2.67	4.03
95% CI	–	(1.17 ~ 2.41)	(1.85 ~ 3.86)	(2.76 ~ 5.88)
*p*-value	–	0.150	0.007	< 0.001
*p* for trend	< 0.001			

Abbreviation: *PRAL* Potential renal acid load, *OR* Odd ratio, *CI* Confidence interval, *Q1* the First quartile, *Q2* the Second quartile, *Q3* the Third quartile, *Q4* the Fourth quartile. *Multiple variates included age, sex, BMI, diastolic blood pressure, systolic blood pressure, smoking, drinking and chronic disease

## Discussion

To our best understanding, this is the first study to explore the association between dietary acid load and hyperuricemia in mainland China. Results from the current study showed a positive association between higher dietary acid load and hyperuricemia. We applied two assessment indicators of dietary acid load including PRAL and NEAP, and the results were consistent. Our results were relative robustness as they were lack of large fluctuations after adjusting a broad of possible risk factors as we could. Since the prevalence of hyperuricemia in Chinese adults is increasing continuously, advocation of well-balanced diet is urgently needed to prevent and control the disease. In addition, we also identified vegetable and fruit consumption were lower in participants with hyperuricemia, but fish consumption higher. Patients with hyperuricemia should eat more vegetable and fruit but less rich-purines foods, such fish, on the premise of dietary diversity.

According to previous reports, high dietary acid load is characterized by both a high consumption of acid precursors and a low intake of base precursors [21]. Acid precursors are mainly from phosphorus and proteins, which have rich sulfur-containing amino acids, while base precursors are largely derived from potassium, magnesium, calcium etc. [23] The aforementioned reveals that high dietary acid load may attribute to a diet pattern with rich in animal-derived food but low in plant-derived food. Taking the diet pattern with high acid load in a long term might change the acid–base balance to acidosis [28]. Even though the potential mechanisms involved in dietary acid load and the risk of hyperuricemia is indeterminate, a previous study indicated that an alkaline urine pH would help excrete uric acid excretion, but not an acidic urine pH [29].

Other studies also exhibited a positive relationship between higher dietary acid load and risk of hyperuricemia. Dayeon et al. [29], found a 21% higher risk for hyperuricemia in middle-aged and older Korean adults with the highest quartile of PRAL, and a 17% higher risk in NEAP according to the Korean Genome and Epidemiology Study [29]. Another cross-sectional study conducted in German and with 6894 adults also identified a significant association between higher dietary acid load and risk of hyperuricemia [30, 31]. The German adults with lower PRAL were found to have a lower odd for hyperuricemia (OR: 0.60; 95% CI: 0.43, 0.83). Our study was in accord with these findings [30, 31]. Even though these studies were observational design, the effect of the higher dietary acid load on chronic diseases cannot be ignored.

There are some limitations in this study which should be notably taken into consideration. Firstly, this study was based on case-control design, which cannot avoid biases, such as recalling bias. Food consumption frequency and amount of some kinds of food might exist deviations with reality or error reporting, in particular in the senior citizens who had impaired memory. FFQ including 12 food groups was applied for food consumption survey, while there must be large bias of the estimation of nutrients intake as the nutrients content of specific foods within the same group may vary greatly, causing inaccurate estimates of nutrients. Secondly, a case-control study could only provide clues to the cause of hyperuricemia and cannot draw a causal conclusion. Thirdly, only adult participants were included in this study, indicative of limited extrapolation of the results. Hyperuricemia is commonly acknowledged as a chronic disease. Even though the age of onset of hyperuricemia trends to be younger, the majority of cases are still among adults, especially those middle-aged and elderly group. Fourthly, the dose-response relationship between dietary acid load and hyperuricemia was less understood. Although we gave *p*-value for trend according to Logistic regression analysis in different group divided by level of dietary acid load, analysis of dose-response relationship was not available as the limited number of participants in this study.

Two aspects should be noted in the future study. The first one is that prospective studies are urgently needed to verify the association between dietary acid load and the risk of hyperuricemia and its possible mechanisms should be also uncovered. The second one is that dose-response relationship between dietary acid load and hyperuricemia should be further studied.

## Conclusion

This study based on case-control design identified a significantly positive association between higher dietary acid load and the odds of hyperuricemia in Chinese adults. Both of PRAL and NEAP had consistent results, indicative of advocation of a well-balanced diet in Chinese population. Given the limitation of case-control study, further prospective studies with large population are urgently needed.

## Data Availability

The data that support the findings of this study are available from the corresponding author upon reasonable request.
